# Transformation of brain myeloid cell populations by SIV in rhesus macaques revealed by multiomics

**DOI:** 10.21203/rs.3.rs-4916594/v1

**Published:** 2024-09-18

**Authors:** Xiaoke Xu, Meng Niu, Benjamin G. Lamberty, Katy Emanuel, Moses Jedd Facun Apostol, Howard S. Fox

**Affiliations:** 1 Department of Neurological Sciences, University of Nebraska Medical Center, Omaha, Nebraska, USA

**Keywords:** single cell, HAND, Microglia, Macrophage, snRNA-seq, snATAC-seq

## Abstract

The primary immune constituents in the brain, microglia and macrophages, are the target for HIV in people and simian immunodeficiency virus (SIV) in nonhuman primates. This infection can lead to neurological dysfunction, known as HIV-associated neurocognitive disorder (HAND). Given the gaps in our knowledge on how these cells respond in vivo to CNS infection, we performed single-cell multiomic sequencing, including gene expression and ATAC-seq, on myeloid cells from the brains of rhesus macaques with SIV-induced encephalitis (SIVE) as well as uninfected controls. We found that the myeloid cell populations were significantly changed by SIVE. In SIVE microglia-like cells express high levels of chemoattractants capable of recruiting highly activated CAM-like cells to the site of infection/inflammation. A unique population of microglia-like cells was found in which the chromatin accessibility of genes diverged from their RNA expression. Additionally, we observed a dramatic shift of upstream gene regulators and their targets in brain myeloid cells during SIVE. In summary, this study further uncovers the transcriptome, gene regulatory events and potential roles of different brain myeloid phenotypes in SIVE.

## Graphical Abstract

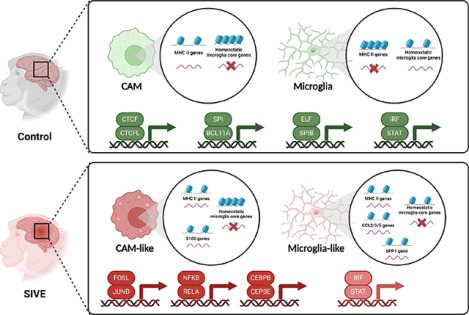

## INTRODUCTION

The human immunodeficiency virus (HIV) is an enveloped retrovirus that contains two copies of a single-stranded RNA genome, which can cause acquired immunodeficiency syndrome (AIDS) by significantly impairing the immune system. HIV remains a global health challenge with profound implications for individuals, communities, and societies. The estimated number of people with HIV (PWH) is 39 million worldwide as of 2022.^1^ According to the latest epidemiology study in 2021, PWH comprise approximately 1.2 million people in the United States.^2^ HIV infection can lead to a spectrum of neurological complications, which are referred to as HIV-associated neurocognitive disorder (HAND).^3^ Clinically, HAND manifests with a range of neurological symptoms and can be classified as asymptomatic neurocognitive impairment (ANI), mild neurocognitive disorder (MND), and HIV-associated dementia (HAD).^4^ Despite advancements in antiretroviral therapy (ART), which have dramatically reduced the prevalence of HAD, there are still 20%−50% of the PWH with milder forms of HIV-associated neurocognitive disorders (HAND).^5,6^ Like HIV in genomic, structural, and virologic perspectives, the simian immunodeficiency virus (SIV) also belongs to the primate retrovirus family. Infection of rhesus macaque by SIV mimics many critical aspects of HIV infection in humans, including immunodeficiency, opportunistic infections, and CNS infection, which can be associated with neurological impairment.^7,8^

The pathogenesis of HAND remains under intense study. One neuropathological link has been HIV encephalitis (HIVE), which is characterized by inflammation of the brain tissue resulting from the direct infection of HIV, as well as secondary effects of viral proteins and immune activation. The exact mechanism by which HIV/SIV infection leads to HIVE/SIVE is still unclear,^9^ but the infiltrating monocytes/macrophages and activated microglia in the brain are thought to be the main contributors.^10–13^ HIV/SIV infection in the CNS is initiated by the entry of infected myeloid and lymphoid cells from the periphery. Once the virus seeds into the brain, the resident myeloid cells (microglia and CNS-associated macrophages (CAM)) could be infected and activated to have enhanced ability in secreting chemokines that can further recruit more activated leukocytes to amplify the infection and inflammation. Several studies have implicated monocyte chemoattractant protein-1 (MCP-1, CCL2) as playing a role in disseminating the virus to the brain through its chemotactic properties on myeloid cells, and elevated levels of this molecule have shown a strong association with HAD.^14–16^ A mutation in the CCL2 gene (encoding MCP-1) that leads to the enhanced infiltration of blood myeloid cells has been reported to be a risk factor for causing HAD.^17^ In addition to CCL2, other chemokines might also be involved in neuropathogenesis caused by HIV/SIV. The activation of resident microglia and macrophages by viral proteins (e.g. gp120)^18–20^ or factors (e.g. cytokines and chemokines)^10,13^ released from infiltrating cells can further damage the neurons and other brain cell types. Additionally, infected microglia and macrophages make up a viral reservoir in the brain under suppressive ART treatment, complicating efforts for an HIV/SIV cure.^21^

In our previous studies, single-cell transcriptomic analyses of brain myeloid cells during different phases of SIV infection uncovered dysregulated genes and cellular phenotypes in acute and chronic infection, the latter in the presence and absence of antiretroviral treatment, as well as end-stage encephalitis.^22–24^ However, using the information on RNA transcripts alone might not be enough to characterize the heterogeneous myeloid populations and comprehensively understand complicated gene regulatory events. Therefore, in this study, we used the single-cell multiomics technique, in which paired single-nucleus RNA sequencing (snRNA-seq) with single-nucleus ATAC sequencing (snRNA-seq). In this manner, we have characterized the myeloid cell phenotypes in the brain to understand the cellular events in those cells during severe CNS infection of SIV (SIVE).

## RESULTS

### SIV-induced encephalitis dramatically changed the brain myeloid cell populations.

**Figure 1.**
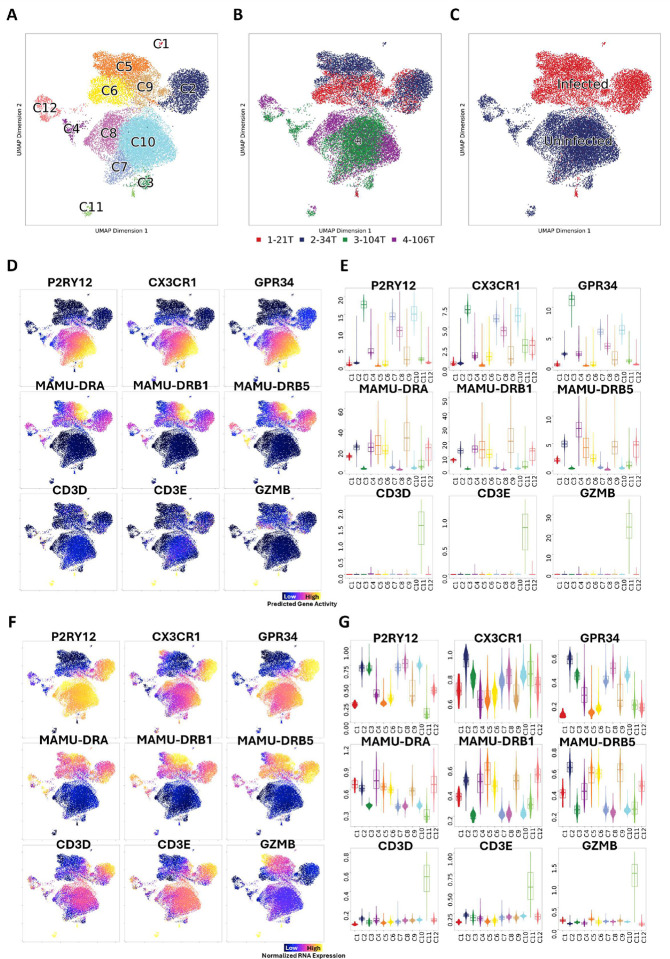
Characterization of different cell phenotypes in the dataset. **(A)** UMAP projection of 24,633 single-cell chromatin and RNA expression profiles, colored by graph-based clustering results **(B)** UMAP projection of single cells in the dataset. The color of each cell indicated the animals that they came from. **(C)** UMAP projection of single cells in the dataset. The color of each cell indicated they were from infected or uninfected animals. **(D and E)** UMAP projection and violin plots of predicted gene activity of known marker genes for microglia, CNS-associated macrophages (CAMs), and lymphocytes. **(F and G)** UMAP projection and violin plots of RNA expression of known marker genes for microglia, CAMs, and lymphocytes.

We used both RNA expression and chromatin accessibility information for cell clustering. In total, we identified 12 different cell clusters (Figure 1A). After quality control, doublet removal, dimensionality reduction, and batch effect correction, the SIVE or control group samples aggregated sufficiently. However, the samples from SIVE animals (21T and 34T) and uninfected animals (104T and 106T) were still separated (Figure 1B), which suggests that the SIVE significantly changed the myeloid cell phenotypes in the normal brain (Figure 1C). After screening the cell markers using snRNA-seq data (Figures 1D **and** 1E) and snATAC-seq data (Figures 1F **and** 1G), we identified microglia, CNS-associated macrophages (CAMs), and a few lymphocytes as the three central cell populations in our dataset.

**Figure 2.**
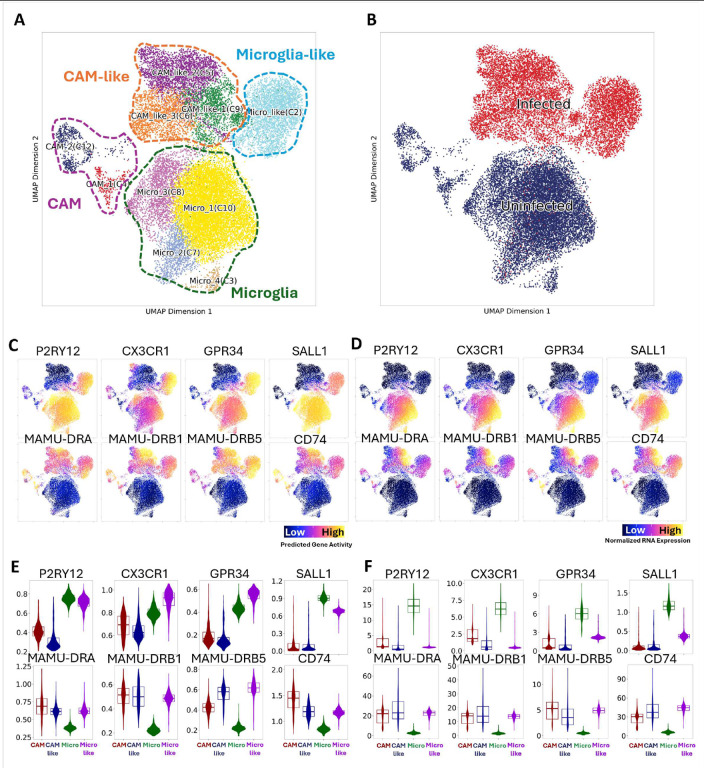
Multiomic characterizations of myeloid cells in the brain of uninfected and SIV-infected animals with encephalitis. **(A)** UMAP projection of 24,420 myeloid-cell chromatin and RNA expression profile, colored by graph-base clustering results. **(B)** UMAP projection was colored by uninfected (blue) and infected (red) status. **(C)** UMAP projection of predicted gene activity of homeostatic microglial core genes and MHC class II molecules. **(D)** UMAP projection of normalized RNA expression of homeostatic microglial core genes and MHC class II molecules. **(E)** Violin plots of predicted gene activity of homeostatic microglial core genes and MHC class II molecules. The cells in CAM, CAM-like, Microglia (Micro), and Microglia-like clusters were aggregated for plotting. **(F)** Violin plots of normalized RNA expression of homeostatic microglial core genes and MHC class II molecules. The cells in CAM, CAM-like, Microglia (Micro), and Microglia-like clusters were aggregated for plotting.

We removed the lymphocyte cluster (C11) and one undefined cluster with only 28 cells (C1) from our dataset. The final dataset included ten myeloid cell clusters (Figure 2A), with six clusters (C3, C4, C7, C8, C10, and C12) found predominately in the uninfected brains, and four (C2, C5, C6, and C9) specifically found in SIVE animals (Figure 2B). The myeloid cells in uninfected brains were different phenotypes of microglia and CAM. The microglial cells were characterized as cells with high expression of homeostatic microglia core genes (e.g. P2RY12, GPR34, CX3CR1, and SALL1) but low expression of MHC class II molecules (e.g. MAMU-DRA, -DRB1, -DRB5, and CD74). Conversely, the CAM were characterized as the cells with high expression of MHC class II molecules but low expression of homeostatic microglial core genes.^25^ The discrepancies between chromatin accessibility (predicted gene activity) and RNA expression for myeloid cells in uninfected brains were minimal (Figures 2C **and** 2D), indicating that the RNA expression of the genes largely reflected their chromatin accessibility in uninfected conditions. As for four SIVE-specific clusters (C2, C5, C6, and C9), we found three of them (C5, C6, and C9) that shared several common findings. For example, they had minimal chromatin accessibility (predicted gene activity) and RNA expression of homeostatic microglial core genes (Figures 2C **and** 2D). Additionally, they were closer to each other but far from the C2 on the UMAP. Distinct from the C5, C6, and C9 clusters, C2 cluster had low RNA expression but high levels of chromatin accessibility of homeostatic microglia core genes, which shared some similarities with microglia in uninfected brains. Given the resemblance between SIVE-specific myeloid clusters and microglia and CAM, we annotated the cells in the C2 cluster as microglia-like cells and those in the C5, C6, and C9 as CAM-like cells. In summary, open chromatin was found for homeostatic microglia genes in both microglia and microglia-like cells (Figure 2E), but there was a divergence in RNA expression (Figure 2F) for those genes between microglia and microglia-like cells. In contrast, CAM and CAM-like cells shared similarities for MHC class II molecules in chromatin accessibility and RNA expression (Figures 2E **and** 2F).

Cluster-specific markers, based on RNA expression **(Figure S1A)** and chromatin accessibility **(Figure S1B)**, were found for each myeloid cell cluster by setting a cut-off of FDR ≤ 0.01 and log_2_FC ≥1.25 **(Table S1)**. The two CAM clusters identified in uninfected controls comprised two different phenotypes that might be derived from two central blood monocyte populations. Classical monocytes have a high capacity for phagocytosis and are efficient producers of pro-inflammatory cytokines, which play a central role in the early stages of the immune response, whereas non-classical monocytes exhibit a patrolling behavior, continuously surveying the endothelial lining of blood vessels for signs of injury or infection.^26,27^ The CAM_1 cluster had higher expression of molecules related to antigen presentation and phagocytosis, whereas the CAM_2 cluster upregulated various adhesion molecules. This suggests that CAM_1 might share an origin with classical monocytes and CAM_2 with non-classical monocytes. The different microglial clusters identified in uninfected brains, while distinct from the CAM clusters, did not show many differences with each other, and they all highly expressed homeostatic microglial core genes. While the CAM-like clusters showed very different transcriptomic profiles and open chromatin regions than microglia-like clusters, the differences between each of the CAM-like clusters were minimal. Therefore, we focused on the differences between CAM-like and microglia-like cells to understand these cell populations in SIVE.

### The gene activity and transcriptomic profile of CAM-like myeloid cells were vastly different from those of microglia-like cells.

**Figure 3.**
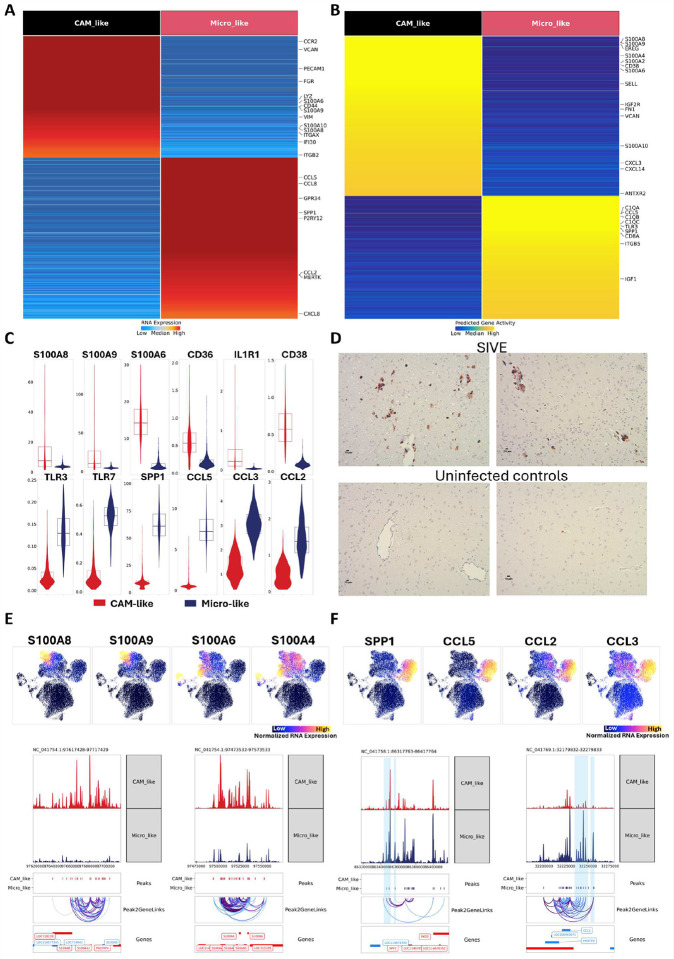
Characterizations of two central myeloid populations in the brains of SIVE animals. **(A and B)** Heatmap of markers for CAM-like and Micro-like (microglia-like) cells using RNA expression or predicted gene activity. The statistical test used for identifying markers was the Wilcoxon rank test, and the cut-off was set as FDR ≤ 0.01 and log_2_ fold change ≥ 1.25. **(C)** Violin plot for RNA expression of selected genes between CAM-like and Micro-like cells. **(D)** Representative images (scale bars, 10μm) of S100A9+ cells in the temporal lobe of two SIVE animals (21T and 34T). The same brain region of two uninfected animals was stained as a comparison. **(E)** UMAP projection of RNA expression of S100A8/9 and S100A4/6 proteins for all myeloid cell clusters. (upper panel) Representative browser plot visualization of pseudo-bulk ATAC-seq, peak-to-gene links (Peak2GeneLinks), and genes at the S100A8/9 and S100A4/6 loci for Micro-like cells and CAM-like cells. (lower panel) **(F)** UMAP projection of SPP1, CCL5, CCL3, and CCL2 RNA expression for all myeloid cell clusters. (upper panel) Representative browser plot visualization of pseudo-bulk ATAC-seq, peak-to-gene links (Peak2GeneLinks), and genes at the SPP1 and CCL5 loci for Micro-like and CAM-like cells (lower panel).

We first compared RNA expression (Figure 3A) and gene activity (Figure 3B) for CAM-like cells and microglia-like cells **(Table S2)**. There were 166 markers in CAM-like cells and 154 in microglia-like cells that were common between RNA expression and chromatin accessibility analyses. Although microglia-like and CAM-like cells were both activated myeloid cells, their upregulated genes differed (Figure 3C). Compared to the microglia-like cells, the CAM-like cells had high RNA expression of molecules in the S100 family (Figure 3C), which have been reported to play critical roles in macrophage signaling. For example, heterodimers formed by S100A8 and S100A9, named calprotectin, serve as an indicator for inflammation.^28,29^ Immunostaining for S100A9 revealed a notable increase in S100A9+ brain myeloid cells in SIVE (Figure 3D). Interestingly, most of the S100A9+ myeloid cells in the SIVE brains are located in the perivascular space, indicating that the CAM-like cells might be activated CAM cells in response to SIVE. In addition, S100A6 and S100A4, which were found to be highly expressed in CAM-like cells, play a vital role in myeloid cell activation through AP-1 signaling. The genomic regions for S100A4 and S100A6 genes are close to each other, as are those for S100A8 and S100A9, and they were all very accessible in CAM like cells but not in microglia-like cells (Figure 3E). Furthermore, Ingenuity Pathway Analysis (IPA), assessing the RNA expression DEGs, revealed that the S100 family signaling pathway was significantly upregulated (p-value: 3.66E10^−14^) in the CAM-like cells. From the gene networks for the S100 family signaling pathway, we found that most of the DEGs in the CAM-like cluster were enriched in S100 proteins that can induce their expression through AP-1 and NF-κB activation **(Figure S2A)**. Some surface markers involved in S100 protein induced NF-κB and AP-1 activation, including CD36, IL1R1, and CD38, were also upregulated in CAM-like cells (Figure 3C). These results suggest that NF-κB and AP-1 signaling, significantly activated by S100 proteins, are critical for CAM-like cells mediated neuroinflammation in SIVE animals. Interestingly the three CAM-like clusters were found to upregulate different S100 proteins. For example, the CAM_like_2 cluster had higher RNA expression of S100A8 and S100A9 proteins, but the CAM_like_3 cluster were found to have higher RNA expression of S100A4 and S100A6 (Figure 3E **and Table S1**).

In contrast, the microglia-like cells were more likely to play roles in immune interactions. The genes encoding complement components (e.g. C1QA, C1QB, and C1QC) and Toll-like receptors (e.g. TLR3 and TLR7) were upregulated in microglia-like cells compared with CAM-like cells (Figures 3A–3C). TLR7 can specifically recognize SIV single-stranded RNA (ssRNA), making it a key sensor for SIV nucleic acids, and both TLR3 and TLR7 can induce the production of type I IFNs and other antiviral factors. The GO and KEGG enrichment analyses **(Figure S2B and S2C)** for RNA markers detected for CAM-like and microglia-like cells further supported that the microglia-like cells had enhanced Toll-like receptor signaling. Some inflammatory molecules were also highly expressed in microglia-like cells but not in CAM-like cells. For example, we found that the SPP1, IL-6, IL-18, and many CCL chemokines had higher RNA expression and chromatin accessibility in microglia like cells (Figure 3C). The chromatin regions for the SPP1 gene were more open, with more marker peaks (FDR ≤ 0.01 & Log_2_FC ≥ 1) found in microglia-like cells compared to CAM-like cells. Interestingly, we found three distal peaks of the SPP1 gene were also open in CAM-like cells, which were highly correlated with SPP1 gene expression (Figure 3F).

The correlations between peak accessibility and gene expression allow for the prediction of specific enhancer-promoter links. In our dataset, we identified 63,855 such potential enhancer-promoter interactions **(Figure S2D)**. Therefore, the correlations between those upstream regions of the SPP1 gene and SPP1 expression suggested they might be the potential enhancers for the SPP1 gene in brain myeloid cells, especially microglia-like cells in SIVE. The CCL chemokines, including CCL5 (RANTES), CCL2 (MCP-1), and CCL3 (MIP-1a), also had high expression in microglia-like cells (Figure 3F) and function as chemoattractants for various immune cells. Like the SPP1 gene, more marker peaks within the CCL5 gene region were found in microglia-like cells. We also found that several peak regions at the CCL5 gene upstream showed strong correlations with CCL5 expression, suggesting the potential enhancer regions. More chromatin accessibility in those enhancer regions coordinated with CCL5 promoter regions, leading to an enhanced expression in microglia-like cells (Figure 3F).

### Microglia-like cells likely play an essential role in attracting other myeloid cells to the site of inflammation and mediating cell-cell interaction during SIVE.

**Figure 4.**
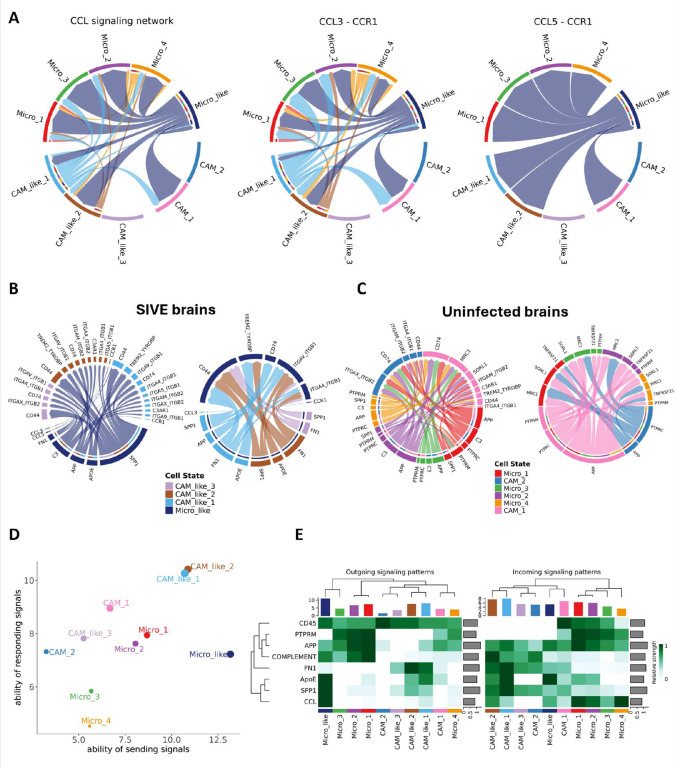
Cellchat analyses for cell-cell communications. **(A)** Chord diagram for visualizing cell-cell communication through CCL signaling pathways, including CCL3-CCR1 and CCL5-CCR1. **(B)** Chord diagram showing the interactions between the myeloid cells mainly found in SIV-infected brains. Left: Signaling sent by microglia-like cells. Right: Signaling sent by CAM-like cells. **(C)** Chord diagram showing the interactions between the myeloid cells mainly found in uninfected brains. Left: Signaling sent by microglial cells. Right: Signaling sent by CAM cells. **(D)** Scatter plot for the dominant senders and receivers for CAM, CAM-like, and Micro-like clusters, based on the total outgoing or incoming communication probability predicted by CellChat. **(E)** Heatmaps for selected signals contributing the most to outgoing or incoming signaling of CAM, CAM-like, and Micro-like clusters. The top-colored bar plot shows the total signaling strength of each cell cluster by summarizing all signaling pathways. The right grey bar shows the total signaling strength of a signaling pathway by summarizing all cell groups.

The initial CNS infection is mediated by the infiltration of peripheral blood leukocytes into the brain, and the subsequent activation of microglia, macrophages, and possibly other cells in the brain could further amplify the neuroinflammation and lead to neurotoxicity. Such inflammation could induce chemoattractant activities and cell-cell interactions of different SIVE-specific myeloid cell clusters. Therefore, we inferred the cell communication probability and cellular communication network for all the myeloid cell clusters identified in this study. Corresponding to the high expression of some CCL chemokines in microglia-like cells, the primary sender in CCL-CCR ligand-receptor interactions, including CCL3-CCR1 and CCL5 CCR1, was microglia-like cells (Figure 4A).

In addition to CCL chemokines, the microglia-like cells interacted with CAM-like cells through SPP1, APOE, APP, and C3-mediated signaling mechanisms. The interaction of SPP1 and CD44, which can promote cell adhesion, migration, and immune responses, seemed most robust between microglia-like cells and different CAM-like cells in the brains with SIVE. While the interactions of microglia-like cells were dominated by the high expression of SPP1, the interaction initiated by the CAM-like cells appeared driven by fibronectin (FN1), well known for involvement in creating extracellular matrices and wound healing (Figure 4B). The myeloid cell interactions in the uninfected normal brain significantly differed from those in the infected brain. Microglial cells in uninfected conditions communicate with CAMs mainly through APP, C3, and PTPRC (CD45), which can interact with CD74, a chaperone for MHC class II molecules on CAMs. The CAMs in uninfected conditions also preferred using APP and PTPRC to communicate with microglia-like cells by binding with MRC1 (CD206) on microglia cells (Figure 4C).

To further understand the involvement of different cell phenotypes in cell-cell interaction, we ranked them based on their ability to send and receive signals. In general, microglia cells in uninfected animals and microglia-like cells in SIVE animals had a stronger ability to send signals than their ability to receive signals, and the CAM cells and CAM-like cells the opposite (Figure 4D). The outgoing and incoming signaling patterns for cell-cell interactions were also very different between the myeloid cells in uninfected conditions and SIVE (Figure 4E). In summary, the cell communication analyses further showed the different functions of microglia-like and CAM-like cells in the brains with SIVE, and the microglia-like cells might be the central phenotype to recruit the inflammatory CAM-like cells to amplify the neuroinflammation.

### Inflammation-related TFs and TF complexes were enriched and highly expressed in SIVE-specific clusters.

**Figure 5.**
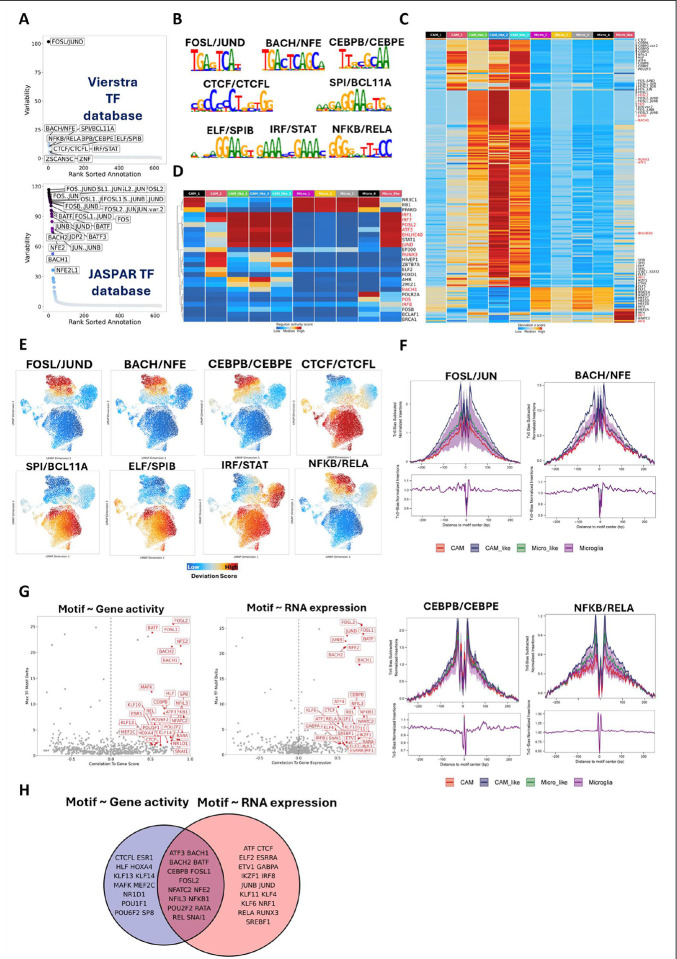
Transcription factor enrichment for different myeloid cell clusters. **(A)** Variability for enriched TF motifs in Vierstra (upper) and JASPAR (lower) databases. The ranking is based on TF motif variability over all accessible chromatin regions. The top 10 TF motifs from the Vierstra database and the top 25 TF motifs from the JASPAR database were labeled. **(B)** The Vierstra archetype consensus of the binding sites for selected high-variable TF complexes. **(C)** Heatmap for motif deviations of TF motifs with an FDR ≤ 0.001 and mean difference ≥ 1 over different cell clusters. The TF motifs were annotated by using the JASPAR database. Top 10 TFs for each cluster were labeled and the TFs that were also predicted by SCENIC (showed in panel D) were labeled in red. **(D)** Heatmap for regulon activity of regulons predicted by SCENIC over different cell clusters. The regulons or TFs that were also found as markers in snATAC motif analyses (panel C) were labeled in red. **(E)** UMAP projection of motif enrichment for selected high-variable TF complexes (annotated by the Vierstra) in different cell clusters. **(F)** The footprints for selected high-variable TF complexes. **(G and H)** Positive TF-regulators whose RNA expression or predicted gene activity positively correlates with the accessibility of their corresponding motif.

To better understand the open chromatin regions of myeloid cell clusters, we performed peak calling on a cell cluster basis. We identified 187,594 peaks from those ten myeloid cell clusters, and more peaks were found in the microglia-like cluster, CAM-like clusters, and Micro_1 cluster compared to other clusters **(Figure S3A)**. The marker peaks (FDR ≤ 0.05 and Log_2_FC ≥ 1) identified in each cell cluster **(Table S3)** further confirmed that SIVE-specific myeloid cell populations had significantly different open chromatin regions compared to microglia and CAMs in uninfected conditions. Next we evaluated the transcription factor (TF) motif enrichment in cluster-specific peaks **(Figure S3B)** and TF activity on a per-cell basis using two TF databases.^30^ We identified some highly variable TF complexes between CAM-like and microglia-like cells, which included FOSL/JUND, BACH/NFE, NFKB/RELA, SPI/BCL11A, CEBPB/CEBPE, ELF/SPIB, CTCF/CTCFL, and IRF/STAT (Figure 5A). The archetype consensus of the binding sites for those high-variable TF complexes is shown in Figure 5B. Including those highly variable TFs, most of marker TFs (FDR ≤ 0.001) enriched in microglia and CAM in uninfected brains were very different from those enriched in SIVE-specific clusters **(Table S4**, Figure 5C **and S3C),** indicating SIVE dramatically shifted the regulatory events in brain myeloid cells. The enrichment of TFs in brain myeloid cell clusters was also predicted by the Single-Cell rEgulatory Network Inference and Clustering (SCENIC) R package^31,32^, where only the RNA expression profile was used to predict the regulon activity in given cell clusters, and yielded fewer enriched TFs, but many in common(labeled in red in Figures 5C **and** 5D).

For highly variable TFs, we found that the FOSL/JUND, NFKB/RELA, BACH (BACH2)/NFE (NFE2), and CEBPB/CEBPE were more enriched in CAM-like cells, IRF/STAT was enriched in microglia-like cells and microglia and CTCF/CTCFL, SPI(SPI1)/BCL11A, and ELF (ELF3)/SPIB were enriched in microglia and CAMs of uninfected animals (Figure 5E, 5F **and S3D**). To confirm the TFs whose motifs were highly enriched in SIVE-specific clusters also had higher expression, we further compare the RNA expression levels and predicted gene activity of those TFs (**Figure S3E-S3H**). The RNA expression and predicted gene activity of most of those TF genes (e.g. NFKB1, NFKB2, RELA, BACH1, BACH2, NFE2, and CEBPB) were positively correlated with their binding motif enrichments, suggesting they might be positive TF regulators. However, the RNA expression and predicted gene activity of STAT1, STAT2, IRF1, and CEBPE were negatively correlated with their binding motif enrichment,^33^ which reflected more complicated regulatory mechanisms. To identify more positive TF regulators whose gene expression or activity is positively correlated with the accessibility of their binding motifs, we performed correlation analyses of TF gene expression or predicted gene activity and their corresponding motif’s enrichment (Figure 5G). The positive TF regulators that were identified by both correlations (Figure 5H) included many TFs mentioned above predominantly upregulated in CAM-like cells (e.g. BACH1, BACH2, NFE2, CEBPB, FOSL1, FOSL2, NFKB1, and REL), highlighting their critical roles in inducing inflammation in this cell type.

Enriched TFs in SIVE-specific clusters also correlated with some inflammatory and anti-viral genes’ RNA expression or predicated gene activity. For example, the RNA expression of IL1β, IL10, and CXCL1 that were regulated by NFKB/RELA (p50/p65) showed a strong positive correlation (r ≥ 0.95) with the motif deviation Z-score of NFKB/RELA **(Figure S4)**. The CAM-like and Microglia-like cells with high enrichment of NFKB/RELA had high expression of IL1β, IL10, and CXCL1. A strong positive correlation was also found for type I IFNs (i.e., IFNα1, IFNα8, and IFNε) and their TFs STAT1/STAT2 **(Figure S4)**. The Microglia-like and Microglia cells with high enrichment of STAT1/STAT2 also had high predicted gene activity of IFNα1, IFNα8, and IFNε. Those results further provide the evidence that the inflammation observed in SIVE-specific clusters was caused by TF shifting in the brain myeloid cells.

### Brain myeloid cell susceptibility to SIV infection is linked to certain enriched cellular pathways and CCR5 expression.

**Figure 6.**
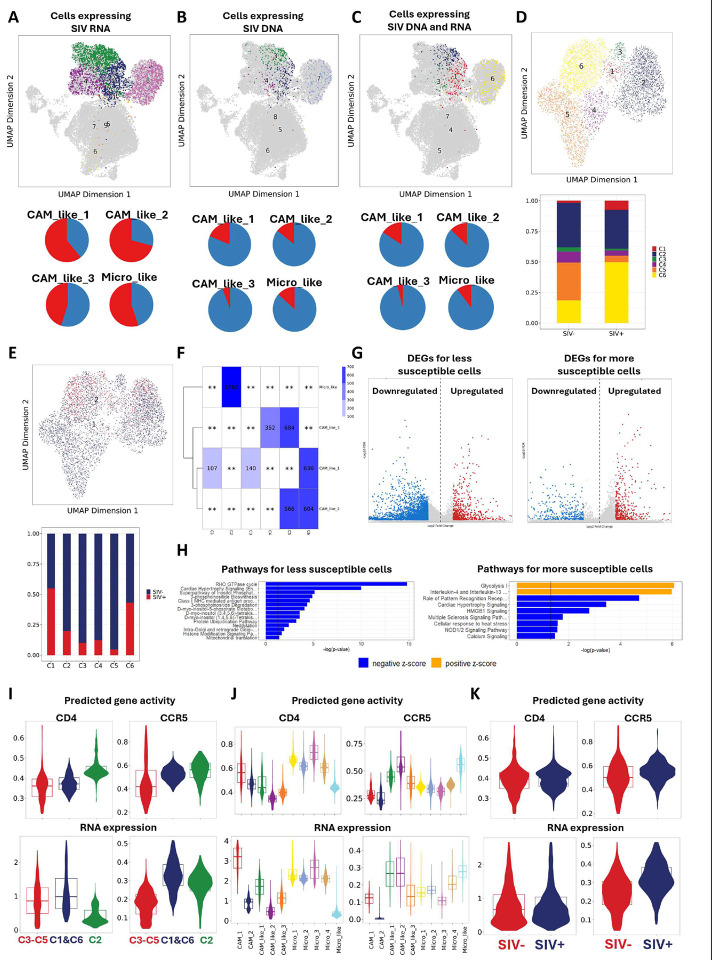
Characterizations of the SIV-infected myeloid cells in the brain. **(A)** UMAP projection of all myeloid cell clusters and the cells detected with SIV RNA were highlighted. The percentage of cells detected with SIV RNA in four SIV-specific clusters was shown in the pie charts. **(B)** UMAP projection of all myeloid cell clusters and the cells detected with SIV DNA were highlighted. The percentage of cells detected with SIV DNA in four SIV-specific clusters was shown in the pie charts. **(C)** The UMAP projection of all myeloid cell clusters and the cells detected with SIV RNA and DNA were highlighted. The percentage of cells detected with both SIV RNA and DNA in four SIV-specific clusters was shown in the pie charts. **(D and E)** The UMAP projection for reclustered cells only included SIV^+^ and SIV− cells in two infected animals. The distribution of SIV^+^ and SIV− cells in the six new clusters was shown in aggregated bar charts. **(F)** Distribution of the cells in new clusters (i.e., C1–C6) and previously defined clusters (i.e., CAM_like_1, CAM_like_2, CAM_like_3, Micro_like). The number of cells was labeled. **(G)** The upregulated marker genes (red) and downregulated marker genes (blue) for the less susceptible clusters (C3–C5) and the more susceptible clusters (C1 and C6). The statistical test used for identifying markers was the Wilcoxon rank test, and the cut-off was set as FDR ≤ 0.01 and absolute log_2_ fold change ≥ 1. **(H)** The top upregulated or downregulated pathways detected for the cells in the less susceptible cluster (left) and the more susceptible clusters (right) by IPA. The cut-off is set as -log(p-value) > 1.3 and |z| > 4.0 for less susceptible clusters and |z| > 2.5 for more susceptible clusters. **(I)** Violin plots of RNA expression and predicted gene activity of CD4 and CCR5 receptors between the more susceptible and less susceptible clusters. **(J)** Violin plots of RNA expression and predicted gene activity of CD4 and CCR5 receptors among all myeloid cell clusters. **(K)** Violin plots of RNA expression and predicted gene activity of CD4 and CCR5 receptors between SIV^+^ cells and SIV− cells.

We added the SIV proviral genome as an additional “chromosome” in the sequence searches to enable the identification of both DNA with accessible open chromatin and RNA gene expression. In the two infected animals with SIVE (i.e. 21T and 34T), SIV DNA sequences (Figure 6A) were identified in 8.9% of the brain myeloid cells in 21T and 15.9% in 34T. More SIV RNA transcripts (Figure 6B) were found in myeloid cells in those two SIV-infected animals: 44.9% in 21T and 67.7% in 34T. Neither DNA nor RNA SIV sequences were found in the two uninfected animals (104T and 106T). However, although ATAC analysis contains more sparse data than RNA analysis, the high expression proportion of SIV-infected myeloid cells raised the possibility of false positives. This is in keeping with the analyses of Plaza-Jennings et al., who examined HIV expression in nuclear preps from brains with HIVE and found that the high expression of HIV in myeloid cells in the encephalitis condition led to contamination of uninfected cells with viral messages, indeed leading to false positives.^34^ Since there were no definitive means to determine which cells truly expressed SIV RNA, we chose a conservative method, requiring both SIV DNA fragments from snATAC-seq and RNA transcripts from snRNA-seq in the same cell to call a cell SIV-positive. (Figure 6C) This restriction for defining SIV-positive cells resulted in 6.7% SIV+ cells in 21T and 13.5% SIV+ cells in 34T. The percentage of SIV+ cells found in 21T and 34T was close to the numbers we previously reported using scRNA-seq (7.6% and 12.8%, respectively) from FACS-sorted intact brain myeloid cells,^23^ which should contain little ambient contaminating RNA, thus giving us confidence in this method.

We then examined the distribution of cells found with SIV RNA and DNA fragments in different myeloid cell clusters. To examine whether there might be a potential myeloid cell phenotype in the brain that was more susceptible to SIV infection, we identified SIV+ cells, defined as above (both DNA+ and RNA+), as well as SIV− cells, which we defined as those without SIV DNA or RNA detected. To eliminate the effect of the SIV viral expression itself on clustering, we excluded the whole SIV genome when we reduced dimensionality and subsequently clustering for this subset dataset. The clustering resolution was set as 0.2, which gave us six cell clusters (Figure 6D **and** 6E). Interestingly, most SIV+ cells were found in C2 and C6 (Figure 6D), but the C2 cluster had even more SIV− cells, which resulted in alow infection rate in this cluster (Figure 6E). On the other hand, we found that over 50% of cells in the C1 cluster were SIV+, although fewer cells were found in this cluster. Given the high infection rate found in C1 and C6 clusters, they were deemed more susceptible to SIV infection. Then we compared these newly identified clusters (i.e. C1–C6) with the previously identified SIVE-specific clusters (i.e. Micro_like, CAM_like_1, CAM_like_2, CAM_like_3) to assess the phenotypes of these clusters (Figure 6F). We found that the cells in the microglia-like cluster were exclusively distributed in the C2 cluster, indicating that the C2 cluster had a microglia-like phenotype, and the other 5 clusters had a CAM-like phenotype. The cells in the CAM_like_1 cluster were clustered in C1, C3, and C6 clusters, and the cells in the CAM_like_2 cluster were clustered in C5 and C6 clusters. In summary, the CAM_like_1, and possibly CAM_like_2, cluster, might be more susceptible to infection.

To better understand what genes might cause the cells in C1 and C6 to be more susceptible to SIV infection, we found the genes that were upregulated and downregulated in the C3–C5 clusters and the C1 and C6 clusters by comparing RNA expression (Figure 6G). Enriching those upregulated or downregulated genes into pathway analyses, we found that the RHO GTPase cycle was downregulated in less susceptible clusters, as were the pathways related to cell activity, including biosynthesis, metabolism, and phosphorylation, (Figure 6H). The more susceptible cells had enhanced glycolysis and IL4 and IL13 signaling, but lower PPR signaling via TLRs and NODs.

We also found that the principal receptors (i.e. CD4 and CCR5) assisting virus entry into the cells were upregulated in the C1 and C6 clusters (Figure 6I), which helps explain the reasons why there were more SIV+ cells found in those two clusters. Additionally, compared to microglia and CAMs found in the uninfected condition, the CAM-like and microglia-like cells found in SIVE downregulated the expression and chromatin accessibility of CD4 (Figure 6J). HIV-induced downregulation of CD4 mRNA (as well as the CD4 protein) has been found in T cells.^35^ When we compared the expression and chromatin accessibility of CD4 and CCR5 in SIV+ cells and SIV− cells in the infected animals, we only found that the RNA expression of CCR5 was slightly higher in SIV+ cells (Figure 6K). Increased expression of the CCR5 protein has been found in microglia and CAM in the brains of those with HIVE.^36^ In total the changes from the uninfected state were likely due to the global effect of inflammation.

### The LTR regions of the SIV DNA found in brain myeloid cells were more accessible than other SIV gene regions.

**Figure 7.**
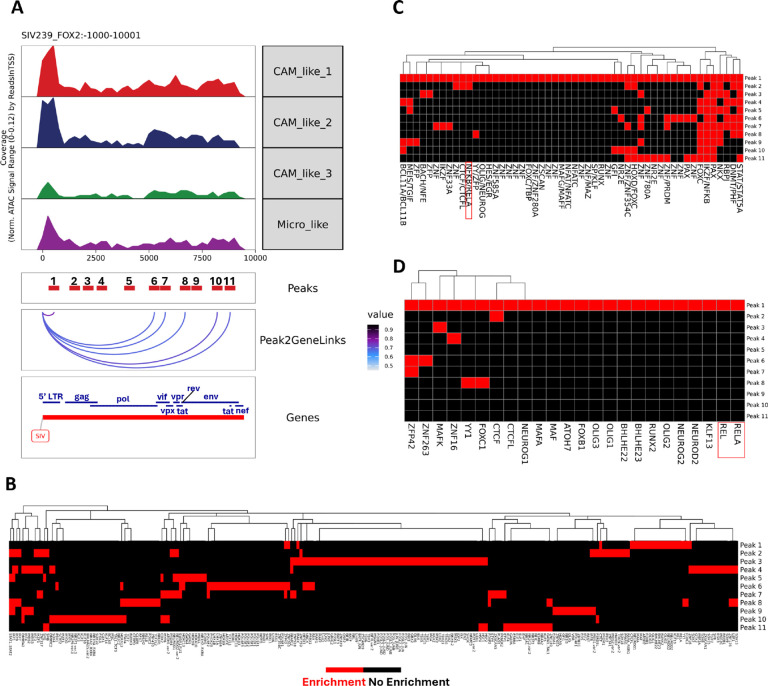
The SIV DNA fragments were aggregated in LTR regions. **(A)** Browser plot visualization of pseudo-bulk ATAC-seq, peaks, peak-to-gene links (Peak2GeneLinks), and annotated SIV gene for all myeloid cell clusters. The SIV fragments were found by mapping to the SIV sequence without 3’LTR, and there were 11 peaks called for SIV gene **(B)** TF enrichment of eleven SIV peaks using the JASPAR database. **(C)** TF complex enrichment of SIV LTR regions using the Vierstra database. **(D)** TF enrichment of SIV LTR regions using the JASPAR database. The TFs in NFKB family were highlighted in the plots for LTR region.

We included the genome of SIV in our annotation as an extra chromosome to identify SIV-infected cells. As discussed in the Methods section, we tested different methods to map the SIV genome. Using the method that kept most SIV fragments, we found that the LTR regions of the SIV DNA were more accessible compared to other regions. (Figure 7A **and S6A**). The more infection-susceptible CAM_like_1 and CAM_like_2 clusters had higher peak signals at LTR regions than CAM_like_3 and microglia-like clusters. The regions encoding Pol, Vif, Vpr, Vpx, and envelope protein (peaks 6, 7, 8, 10, and 11) also showed more accessibility and a strong association with the SIV RNA expression (Figure 7A).

To understand what host TFs myeloid cells might use for SIV transcription, we performed TF motif enrichment specifically for the SIV peaks. While Peak 3 (coordinate: 1891–2391) had a large number of enriched motifs, with most related to FOS/JUN (Figure 7B), in the LTR there was enrichment for transcription factors related to NF-κB (e.g. REL, RELA, NFKB/RELA), which were very specific for the LTR region (Figure 7C **and** 7D). This further validates that the SIV in the infected brain myeloid cells could use host NF-κB TFs to express its genes, as previously reported.^37–39^

### Neuroinflammation in animals with SIVE might further differentiate myeloid cells toward the CAM-like phenotype.

Given the plasticity nature of myeloid cells in response to the inflammation, we performed the trajectory analyses to find the differentiation hierarchy and the potential genes and TFs driving the differentiation. We first used unsupervised trajectory analyses for pseudo-time prediction. The homeostatic microglia cluster found in uninfected conditions and with most microglia (Micro_1) was set as the root node **(Figure S5A)**. The cells in the CAM_like_2 and microglia-like clusters had the highest pseudo-time values, indicating they might be at the late stage of differentiation. The end of the trajectory path with Micro_1 as the root node pointed to microglia-like cells but not CAM-like cells. We then set CAM_1, the classical macrophage population in the uninfected brain, as the root node for prediction **(Figure S5B)**. Again, the end of the trajectory pathway pointed to microglia-like cells but not CAM-like cells, which suggests CAM-like cells might not arise from microglia or CAMs found in uninfected brains. In addition, we found that the gene expression and predicted gene activity for the CEBPB gene, which becomes active during the differentiation process, were higher in CAM-like cells compared to microglia-like cells **(Figure S5A and S5B)**, indicating CAM-like cells might be more polarized than microglia-like cells. Therefore, it is possible that suppressing the transcription of homeostatic microglial core genes. However, the chromatin accessibility of homeostatic microglial core genes did not decrease at this stage of differentiation (Figures 2D **and** 2F). With the progression of neuroinflammation, it is possible that some activated microglia-like cells might further differentiate into CAM-like cells, which completely shut down the chromatin accessibility for homeostatic microglial core genes and show more pathogenic.

**Figure 8.**
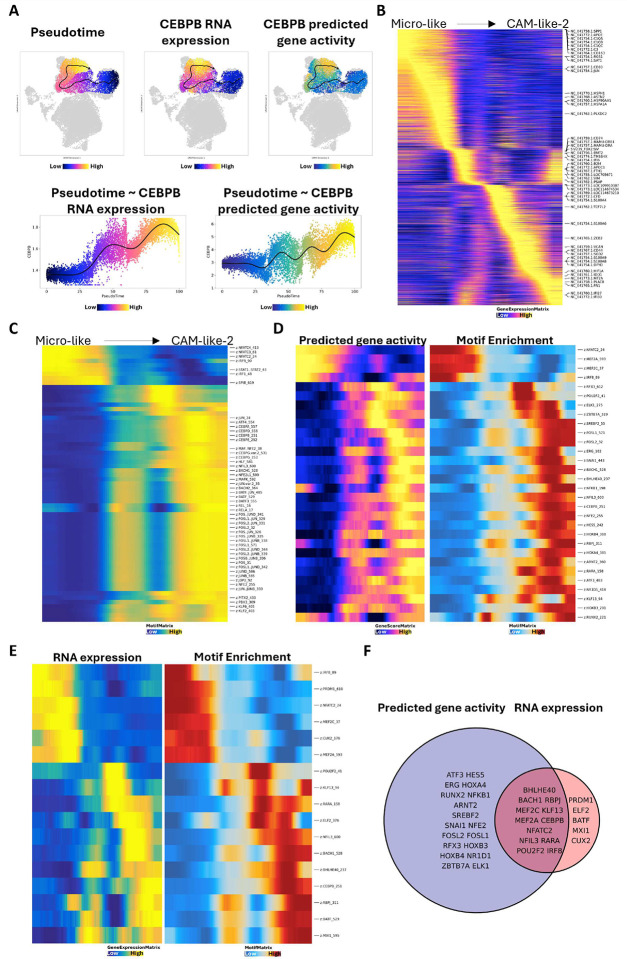
Identifying the genes and transcription factors driving the differentiation of brain myeloid cells during SIVE. **(A)** The pseudo-time for the brain myeloid cells was mainly found in SIVE animals (left), and the correlation between cell pseudo-time and predicted gene activity (middle) or RNA expression (right) of the CEBPB gene. **(B)** The changes of the genes regarding their RNA expression across pseudo-time. **(C)** The changes of the transcription factors (TFs) regarding their motif enrichment across pseudo-time. **(D)** The positive TF regulators with high predicted gene activity and motif enrichment across pseudo-time. **(E)** The positive TF regulators with high RNA expression and motif enrichment across pseudo-time. **(F)** The positive TF regulators were identified by both correlations (shown in D and E), which might drive the differentiation of brain myeloid cells in SIVE.

Based on this hypothesis, we performed trajectory analyses only for the SIVE-specific clusters (Figure 8A). The trajectory pathway started from microglia-like cells and ended in the CAM_like_2 cluster, which was found to be more pathogenic in the above analyses. The predicted pseudo-time for the SIVE-specific clusters positively correlated with the RNA expression and chromatin accessibility (predicted gene activity) of the CEBPB gene (Figure 8A), confirming that this differentiation path is reasonable. Then, we further visualized the changes in RNA expression (Figure 8B), chromatin accessibility **(Figure S5C)**, peaks **(Figure S5D)**, and motif enrichment (Figure 8C) across this differentiation. From the RNA expression changes (Figure 8B), we found that the differentiation process decreased the pathogen recognition and alarm responsiveness of the brain myeloid cells since the RNA expression for complements and scavenger receptors was absent in CAM-like cells. The RNA expression of heat shock proteins was also downregulated during the differentiation from microglia-like cells to CAM-like cells. On the other hand, this differentiation leads to enhanced expression of MHC class II molecules, S100 proteins and interferon-inducible proteins. From the changes in enriched TFs (Figure 8C), the differentiation shifted the motif enrichment from STAT and NFATC families to CEBP, BATF, FOS/JUN, and KLF families. The rise in binding sites for inflammatory-linked TF found in end-differentiated cells suggested that the differentiation of myeloid cells from microglia-like to CAM-like is accompanied by increasing regulatory events for genes contributing to inflammation. To better understand the TF regulation during this differentiation/activation, we correlated the RNA expression or predicted gene activity (chromatin accessibility) with the TF motif accessibility across pseudo-time. We identified the positive TF regulators that might drive this differentiation process (Figure 8D **and** 8E). Many common positive TF regulators were potentially found to drive this differentiation (Figure 8F). For example, IRF8, NFATC2, MEF2A, and MEF2C had higher RNA expression, predicted gene activity, and motif accessibility in the cells at an early stage of differentiation, suggesting the activity of those TFs might related to the polarization of microglia-like cells to CAM-like cells. However microglia-like cells and CAM-like cells may be from very different progenitor cells that are not interchangeable. Such cells may come from blood myeloid cells that enter the brain in inflammatory conditions such as SIVE. It is also possible that CAM-like cells and microglia like cells can be polarized to each other, responding to different inflammatory milieu.

## DISCUSSION

Myeloid cells in the brain are essential in inducing neurocognitive disorders once activated. In HIV infection, the invading virus can reshape the immune milieu in the brain, which further restructures the myeloid cell constitution. This study found that the myeloid cell phenotypes in the brain with SIVE significantly differed from those in the uninfected brains Given the advantages of multiomic sequencing, we found four different phenotypes in the infected encephalitic brain, broadly classified into two primary phenotypes: microglia-like and CAM-like. The CAM-like myeloid cells were more pathogenic and wholly lost the expression and chromatin accessibility of homeostatic microglial core accessibility genes. On the other hand, microglia-like myeloid cells suppressed the RNA expression of homeostatic microglialcore genes but surprisingly kept the chromatin accessibility of those genes.

By assessing the microglia-like and CAM-like phenotypes using snRNA-seq and snATAC-seq information, we found that the microglia-like cells (relative to microglia cells) had increased RNA expression and chromatin of SPP1 and chemokines, and CAM-like cells (relative to CAM cells) upregulated S100 proteins. Cell communication analyses further revealed the potential biological consequences for upregulating SPP1 and chemokines in microglia-like cells. The SPP1 highly expressed in microglia-like cells was likely to bind with the CD44 on CAM-like cells. The interaction between SPP1 and CD44 has been widely reported in cancer for immune cell infiltration^40,41^, and those studies also highlighted the ability of SPP1-CD44 interaction to modulate cell adhesion and movement. Combining the facts that chemokines also actively induce infected/activated myeloid and lymphoid cell migration in HIV infection,^42,43^ the microglia-like phenotype might be the main initiator of inflammatory cell chemotaxis in the brain with SIVE. CCL3 and CCL5 are natural ligands for the primary HIV-1 coreceptor CCR5, which may prevent HIV from entering cells.^44,45^ Thus, microglia-like cells might also contribute to preventing SIV infection by secreting those two chemokines. The CAM-like cells might possess a completely different biological function in SIVE.^29^ Although S100A8/A9 is reported to suppress HIV replication in macrophages,^46^ their ability to promote inflammatory conditions such as lethal endotoxin-induced shock^29^ can lead to brain damage. The other S100 proteins were also found to have higher expression in CAM-like cells but not microglia-like cells, and their function is to serve as alarmins to induce and amplify the immune response through degranulation.

The TF motif enrichment in CAM-like and microglia-like cells further indicated their differences. Although most TFs enriched in SIVE-specific clusters were significantly different from TFs enriched in CAM or microglia, the CAM-like cells were enriched with much more inflammatory TF motifs than microglia-like cells. The TFs in the IRF/STAT family enriched in microglia-like cells were also enriched in microglial clusters (found in uninfected brains) but were barely enriched in CAM-like cells. Correspondingly, some type I IFNs were also highly expressed in microglia-like and microglia cells but not CAM-like cells. The TFs in the IRF/STAT family regulated the expression of type I interferons, essential for anti-viral activity.^47–49^ Additionally, the anti-inflammatory properties of type I IFNs^50–52^ further suggested the potential immunoregulatory roles of microglia-like cells in SIVE brains. On the other hand, the CAM-like cells were highly enriched with FOS/JUN (AP-1) and NFKB/RELA, the TF complexes mediating immune activation in brain myeloid cells.^53,54^

While the microglia-like and CAM-like cells appeared activated to defend against the invading virus in SIVE, many infected myeloid cells could still be found in the brain. Our estimation based on SIV RNA transcripts and DNA fragments indicates that the infection rate in brain myeloid cells was ~ 10% in SIVE. Furthermore, we found that some cells in CAM-like clusters, mainly in the CAM_like_3 cluster, were less likely to be infected. However, other CAM-like cells were more likely to be infected, mainly in the CAM_like_1 and CAM_like_2 clusters. By assessing the upregulated and downregulated genes and pathways between more susceptible and less susceptible brain myeloid cells in SIVE, we found that the less susceptible cells significantly downregulated many cellular activities. However, we still do not know if the suppressed cellular activities caused the lower susceptibility to SIV infection or if the lower levels of SIV infection led to those cells being comparatively less active. Regarding infection, the more susceptible cells upregulated the expression of CCR5 but not CD4. Both CD4 and CCR5 can assist the HIV/SIV entering the cells, but their changes in response to SIV infection were very different (Figure 6J). The downregulation of CD4 has been widely reported during HIV infection, and this downregulation is thought to be caused by Nef proteins for stimulating HIV-1 production and infectivity.^55–57^Upregulation of CCR5 may be important in susceptibility to infection because it serves as a critical receptor for HIV and SIV infection, and its modulation correlated with infectability.^58,59^

The advantages of ATAC-seq inclusion enabled us to understand SIV DNA in the host nucleus. Most of the accessible chromatin found in the SIV DNA were at the SIV long terminal repeats (LTRs), which are essential regions for viral gene expression and latency. The proviral genomes of HIV are flanked by two LTRs. Each LTR consists of three regions: U3, R, and U5.^60^ Among those LTR regions, the U3 region contains promoter, enhancer, and modulatory elements that regulate the expression of viral genes. The promoter region contains three Sp1 binding sites, and the enhancer region contains two NF-κB and one NFAT binding site^61–64^, which were also enriched at the SIV LTR region in our dataset. We also found more de novo host TF binding sites that have not been reported, and their potential involvement in modulating HIV/SIV gene expression needs further investigation. In addition to the LTR, five additional peaks within the SIV genome identified by snATAC-seq were strongly correlated with expression. As open chromatin is more accessible for modification by methods such as CRISPR-Cas9, this may provide new regions to target to shut down or eliminate HIV/SIV from myeloid cells.

In conclusion, the high-throughput multiomic sequencing technique highly promotes the identification of heterogeneous myeloid cell populations in uninfected brains and brains with SIVE. Including simultaneous snATAC-seq with snRNA-seq enabled a deeper understanding of the gene regulatory events in brain myeloid cells, especially during SIVE. However, due to the many considerations for the investigations related to NHPs, the results and conclusions in this study, which were generated from a limited number of SIVE animals, will need further study. The deposition of sequence data and metadata in publicly accessible databases from our studies and others enables the building of larger analyses with more subjects. These data can be useful in meta-analyses across models and disease states.

## MATERIALS AND METHODS

### Animals

The four male adult rhesus macaques used in this study tested negative for the indicated viral pathogens: SIV, SRV, STLV1, Herpes B-virus, and measles; and bacterial pathogens: salmonella, shigella, campylobacter, yersinia, and vibrio. Macaques were housed in compliance with the Animal Welfare Act and the Guide for the Care and Use of Laboratory Animals in the NHP facilities of the Department of Comparative Medicine, University of Nebraska Medical Center (UNMC). The American Association for Accreditation of Laboratory Animal Care International has accredited the primate facility at UNMC. The UNMC Institutional Animal Care and Use Committee (IACUC) reviewed and approved this study under protocols 19-145-12-FC and 16-073-07-FC. Animals were maintained in a temperature-controlled (23 ± 2° C) indoor climate with a 12-h light/dark cycle. They were fed Teklad Global 25% protein primate diet (Envigo, Madison, WI) supplemented with fresh fruit, vegetables, and water ad libitum. The animal care and veterinary personnel observed the monkeys twice daily to check their health status. Two of the four animals (21T and 34T) were intravenously inoculated with a stock of SIVmac251 and developed SIV-induced encephalitis (SIVE). The other two macaques (104T and 106T) were uninfected and used as controls. Results from scRNA-seq of microglia for 21T (who had been treated with methamphetamine) and 34T have been previously reported.^22^.

### Viral loads

To determine the terminal viral load in plasma, the blood of infected animals (21T and 34T) was collected. The EDTA-anticoagulated plasma was separated from blood by centrifugation. Brain and lymphoid organ specimens were taken to determine viral load in tissues. Plasma and tissue SIV RNA levels were determined SIV RNA was measured using the branched DNA assay by Siemens (Emeryville, CA). The viral load in plasma, lymphatic tissues, and brain of those two animals could be found in our previously published paper.^22^

### Immunohistochemical staining

Brains were fixed in 10% neutral buffered formalin, embedded in paraffin, cut into 5 μm sections, and mounted on glass slides. For immunohistochemistry, sections were rehydrated, and endogenous peroxidase activity was blocked by a 3% hydrogen peroxide treatment in absolute methanol. Following that, a heat treatment with 0.1 M citrate pH 6.39 was performed for antigen exposure. Sections were blocked with 5% Normal Horse Serum (Vector Labs, Burlingame, CA, USA) in PBS and incubated with the primary antibody diluted in the same buffer. Antibodies were targeted against S100A9 (Cat# PA5–79949, dThermo Fisher Scientific, Rockford, IL, USA) at a 1–10,000 dilution. Biotinylated secondary antibodies (horse anti-rabbit IgG Cat# MP-7401, Vector Labs, Burlingame, CA, USA) were used. Visualization was achieved using Nova Red (Vector Labs, Burlingame, CA, USA). Counterstaining was done using Gill 2 Hematoxylin (StatLab, McKinney, TX, USA).

### Isolation of myeloid cells in the brain

92 days (for 21T) and 49 days (for 34T) after viral inoculation, a necropsy was performed due to symptoms in the animals consistent with simian AIDS. Uninfected animals 104T and 106T were necropsied per approved animal protocol. Animals were deeply anesthetized with ketamine plus xylazine and blood cleared from the brain and other organs by intracardial perfusion with sterile PBS containing 1 U/ml heparin. Brains were harvested, and approximately half of the brain was taken for microglia/macrophage isolation. Microglia/macrophage-enriched cellular isolation was performed using our previously described procedure.^65^ Briefly, the brain was minced and homogenized in cold Hank’s Balanced Salt Solution (HBSS, Invitrogen, Carlsbad, CA). After being centrifuged, the brain tissue was digested at 37° C in HBSS containing 28 U/ml DNase I and 8 U/ml papain for 30 minutes. After digestion, the enzymes were inactivated by adding 2.5% FBS, and the cells were centrifuged and resuspended in cold HBSS. The cell suspension was mixed with 90% Percoll (GE HealthCare, Pittsburg, PA) to a final concentration of 20% Percoll and centrifuged at 4° C for 15 minutes at 550 × g. The microglia/macrophage pallet at the bottom was resuspended in HBSS and passed through a 40 μm strainer to remove cell clumps and aggregates. Cells were again pelleted by centrifugation and resuspended in RBC lysis buffer for 3 minutes to eliminate contaminating red blood cells. A final wash was performed before the resulting cells were quantified on a hemocytometer and Coulter Counter Z1. The cells were resuspended in 10% DMSO, 90% fetal bovine serum and subjected to slow controlled freezing followed by storage in liquid N_2_. The methods for cryopreservation followed our previous study, which was found to maintain the vast majority of the transcriptomic features of fresh isolated microglia/macrophages.^65^

### Single nuclei preparation and multiomic sequencing (ATAC and gene expression)

Cryopreserved cell isolates were rapidly thawed in a 37° C water bath. The cell recovery procedures were well described in our previous publications.^65^ After the recovery, cells were washed and counted by Coulter Counter Z1. Once cell concentration was known, cells were transferred to ice-cold PBS and stained with CD11b (Biolegend 101257) and UV-blue live/dead (Invitrogen L23105). Cells were washed, resuspended in flow cytometry staining buffer (e-bioscience), and sorted on an Aria2 flow cytometer (BD Biosciences, San Jose, CA, USA).

Nuclei were isolated from microglia cells using the 10XGenomics protocol. Nuclei were then quantified on a hemocytometer and concentrated to approximately 2200–2400 nuclei per μL. Based on 10× Genomics parameters targeting 8000 nuclei, the ideal volume of cells was loaded onto the 10× Genomics (Pleasanton, CA, USA) Chromium Next GEM Chip J and placed into the Chromium Controller for nuclei capturing and library preparation. The prepared libraries were sequenced using Illumina (San Diego, CA, USA) Novaseq6000 sequencers. The sequences have been deposited in NCBI GEO (accession number GSE272669).

### Pre-processing of multiomic data

Sequenced samples were processed using the 10× Genomics Cell Ranger ARC pipelines (2.0.2). Specifically, the multiomic data was demultiplexed and aligned to a customized genome combining the rhesus monkey reference genome (NCBI RefSeq assembly Mmul_10) and an SIV genome that we constructed by sequencing our virus stock (NCBI GenBank, accession number PP236443). Since SIV had two LTR regions with repeating sequences and the Cell Ranger arc count pipeline discarded the reads mapped to two identical regions (MAPQ mapping score < 30), we built three testing reference genomes containing the same rhesus macaque genome but different SIV genome (i.e., remove 3’ LTR region, remove 5’ LTR region, and keep the whole length of SIV genome) for Cell Ranger ARC count to test on two infected samples. After comparing the percentage of reads mapped to the SIV genome but with low MAPQ mapping scores (<30) and the number of SIV DNA fragments in fragment files generated by Cell Ranger among the three tests, we found that removing 3’LTR and keeping 5’LTR regions allowed minimized the low-quality SIV reads **(Figure S6B)** and maximized the SIV DNA fragments **(Figure S6C)** to pass the filtering of Cell Ranger ARC count. Therefore, we decided to use the genome containing the Mmul10 rhesus macaque genome and the SIV genome without 3’LTR for aligning and counting all the samples. After the Cell Ranger ARC count pipeline, the filtered feature barcode matrices containing the RNA count and fragment files containing the ATAC fragment count were obtained. The counting summary statistics generated by 10x Genomics for each sample are shown in **Table S5**.

### Characterization of cell phenotypes in muti-omics dataset

Pre-processed ATAC-seq data from multiomic sequencing processed with Cell Ranger arc were read with the ArchR R package (version: 1.0.2). We built our own gene and genome annotation used in ArchR by the BsgeomeForge R package and the GenomicFeature R package, and the input files for those two packages were the gtf and fa files that were used to build the reference genome in Cell Ranger. After successfully reading the fragment files, we performed several steps of quality controls for the cell barcodes in the fragment files. Firstly, the cell barcodes were filtered based on the ATAC data, and the cell barcodes with less than 1000 fragments per cell and a TSS enrichment score of less than four were removed. Then, we integrated ATAC-seq data with filtered RNA count matrices by using the import10xFeatureMatix function in the archR package, and the cell barcodes with both RNA data and ATAC-seq data were retained. After integration, we filtered the cell barcodes based on the RNA data. The cell barcodes with gene count and UMI count of less than 400 and mitochondria percentage of more than 15% were removed. Finally, we identified and removed the inferred doublets using addDoubletScore and filterDoublets functions in archR. After filtering, 24,633 cells were left for downstream analyses. The QC plots showed that all four samples had a similar distribution of their TSS enrichment score, number of unique fragments, UMI counts, and gene counts **(Figure S7A and S7B)**. After quality control, dimensionality reductions were performed separately using the iterative Latent Semantic Indexing (LSI) approach for RNA and ATAC data. The parameters set for implementing LSI in archR were iterations = 2, res = 0.2, maxClusters = 6, varFeatures = 25000 (for ATAC), varFeatures = 2500 (for RNA) dimsToUse = 1:30, LSI method = log(tf-idf), and the other unmentioned parameters followed the default. The addCombinedDims function in archR combined the dimensionality reductions from ATAC and RNA. The batch effect between different samples was addressed using the Harmony implementation of archR. Then, clustering was performed using dimensionalities combining RNA and ATAC data, and the resolution was set at 0.2. Finally, UMAP dimensionality reduction was performed to visualize data, and again, the combined dimensionalities were used. The following characterizations of each cell cluster were performed in two ways. Firstly, we screened the normalized RNA expression and predicted gene activity of the gene markers for microglia (e.g. P2RY12, GPR34, CX3CR1), CNS-associated macrophages (e.g. MAMU-DRA, MAMU DRB1, MAMU-DRB5), and lymphocytes (e.g. CD3D, CD3E, GZMB). The predicted gene activity was the gene scores estimated by archR based on chromatin accessibility information. The predicted gene activity scores and RNA expression were imputed for visualization using MAGIC to smooth signals across nearby cells. Another way to visualize chromatin accessibility is through cluster-based genome browser tracks using the plotBrowserTrack function. In the visualization of chromatin accessibility for the SIV gene, the parameters for plotting were set as upstream = 500, downstream = 10000, and for other genes the parameters were set as default. After the initial screening, we found some lymphocyte contaminations, which were removed from our analyses. The marker genes were subsequently identified in only myeloid cell populations using normalized RNA expression and predicted gene activity **(Table S1)**. The Wilcoxon statistical test was implemented by the getMarkerFeatures function in archR, and the marker genes were then identified by setting the cut-off as FDR ≤ 0.01 and log2 fold change ≥ 1.25. The marker genes were plotted in a heatmap using the markerHeatmap function in arhcR for visualization.

From the UMAP projection and initial characterization, we found two central myeloid populations in the infected brains. Given their RNA expression and predicted gene activity profiles, we annotated the two populations as CAM-like cells and microglia-like (Micro-like) cells. To find their differences, we subset those cells and merged each population’s subclusters (i.e., CAM_like_1, CAM_like_2, and CAM_like_3) into one cluster to compare their RNA expression and predicted gene activity. As mentioned, the statistical test used for identifying markers was the Wilcoxon rank test, and the cut-off was set as FDR ≤ 0.01 and log_2_ fold change ≥ 1.25. To better understand those changes, we further enriched the CAM like and microglia-like cell markers into Gene Ontology (GO) pathway analyses (Biological Process, BP was selected), Kyoto Encyclopedia of Genes and Genomes (KEGG) pathway analyses, and Ingenuity Pathway Analyses (IPA). The GO and KEGG analyses were implemented by the clusterProfiler R package (version 4.0.2).^66^

### Cell-to-cell communication analyses

The cell-cell communication network’s inference, analyses, and visualization were implemented in the CellChat v2 R package (version 2.1.0).^67,68^ To obtain the input file for CellChat analyses, we preprocessed the filtered feature barcode matrix from each sample in the Seurat R package (version 4.4.0).^69^ We first created and merged the Seurat objects of different samples, and then we removed the cells not included in the ArchR object by matching the cell barcode. After this filtering, all the cells in the ArchR object were also included in the Seurat Object. In addition, the annotation for each cell was also transferred from the ArchR object to the Seurat object. Then, we normalized the RNA counts using the log normalization method in Seurat, and the log-normalized data was further used to create a CellChat object. The ligand-receptor interaction database provided by CellChat only includes the mouse and human, so we selected the human database, which is more relative to rhesus monkeys for prediction. After subsetting signaling genes and identifying over expressed ligand-receptor interactions among cell clusters, the computeCommunProb function in the CellChat computed the communication probability. We selected the “trimean” method to compute the communication probability, which produced fewer but stronger interactions. We obtained the communication probability on the signaling pathway level by summarizing the communication probabilities of all ligand-receptor interactions associated with each signaling pathway, and this was achieved by the computeCommunProbPathway function. Finally, we used the aggregateNet function to calculate the aggregated cell-cell communication network. To visualize the involvement of different cell clusters in the CCL signaling pathway, the chord diagram was used, and the cells were grouped into four categories, including microglia, CAM, microglia-like, and CAM-like. We also used the chord diagram to visualize the interactions of myeloid cells in uninfected and SIV-infected brains. We grouped the cells into CAM and microglia for uninfected brains and CAM-like and microglia-like for SIV-infected brains. Then, we plotted all the interactions sent from either of the two groups to another group for uninfected brains and SIV-infected brains separately. Then, we identified the dominant senders and receivers in the intercellular communication network using the netAnalysis_comuteCentrality function, and the total outgoing or incoming communication probability associated with each cell cluster was shown in a 2D scatter plot.

### Peak Calling and transcription factor (TF) motif enrichment

Single-cell chromatin accessibility data were used to generate the pseudobulk replicates by the ArchR function, addGroupCoverages, for peak calling with MACS2.^70^ The implementation of MACS2 in the ArchR was through the addReproduciblePeakSet function, in which we set genome size as 2.2e9, which, as suggested by MACS2 documentation, is 78% of the total genome size of rhesus macaques.^71^ The pseudobulk replicates and peak calling were based on the ten myeloid cell clusters identified in this study. Marker peaks for each myeloid cell cluster were further found using the getMarkerFeatures function on the peak matrix embedded in the ArchR object. The statistical test used for finding marker peaks was the Wilcoxon rank test, and the cut-off was set as FDR ≤ 0.01 and log_2_ fold change ≥ 1. Then, we determined the enrichment of transcription factor binding sites for the marker peaks by the peakAnnoEnrichment function. Before the enrichment, we used two human TF motif databases, including the JASPAR 2020 database and the Vierstra non-redundant TF (version: 2.1) database,^30^ to annotate the peaks. We extracted the binarized motif enrichment information for 11 SIV peaks using the getMatches function in ArchR to understand the host TF enrichment for SIV DNA fragments. We used the chromVAR R package^72^ embedded in ArchR to calculate the TF enrichment per-cell basis. A background peak set controlling for total accessibility, and GC-content was generated by the addBgdPeaks function before ChromVAR was run with the addDeviationsMatix function, using JASPAR or Vierstra motif set to calculate enrichment of chromatin accessibility at different TF motif sequences in a single cell. The motif deviation z-scores were used to find marker motifs in each myeloid cell cluster and for visualization after being imputed with MAGIC. The statistical test used for finding marker motifs was the Wilcoxon rank test, and the cut-off was set as FDR ≤ 0.001 and MeanDiff ≥ 1. Transcription factor footprinting was performed and visualized using the ArchR functions, which included getFootprints and plotFootprints (normMethod = “subtract,” smoothWindow = 6).

### SCENIC analysis

SCENIC (version 1.1.2–01) (Single-Cell Regulatory Network Inference)^31,32^ was to examine the transcription factors controlling the gene regulatory networks in the identified cell clusters. This method examines the coexpression of genes with transcription factors followed by *cis*-regulatory motif analysis to identify significant motif enrichment of the correct upstream regulator, termed “regulons.” Gene expression RNA counts for each cluster were used as input, and analysis was conducted on enriched regulons. SCENIC uses a random forest^73^ to calculate the importance of each regulon found in the expression matrix. After being ranked, the top 50 regulons annotated by RcisTarget, were selected and activity scores for regulons were calculated using AUCell. Regulons with less than 20 genes and low confidence (termed “extended”) regulons were filtered out.

### Integrated chromatin accessibility and RNA expression

As mentioned, the DNA fragments and RNA transcripts from multiomic sequencing were initially integrated. We performed two integrative analyses involving the features’ correlation to predict the gene regulatory interactions. Peak-to-gene links (PGLs) were calculated by correlations between peak accessibility and snRNA expression data using the addPeak2GeneLinks function. The dimensionality that combined ATAC and RNA information after batch effect correction was used, and other parameters were set as default for PGLs. All PGLs were plotted in a peak-to-gene heatmap using the plotPeak2GeneHeatmap function for visualization. We also identified positive TF regulators by correlating the gene expression or predicted gene activity of TFs and the accessibility of their corresponding motifs. This analysis started by identifying deviant TF motifs using the getGroupSE function, then correlating TF motifs and TF gene expression or predicted gene activity using the correlateMatrices function. The positive TF regulators were considered as TFs whose correlation between motif and gene expression (or predicted gene activity) is more than 0.5 with an adjusted p-value less than 0.01 and a maximum inter-cluster difference in deviation Z-score that was in the top quartile. The TF motif database used for these correlations was JASPAR. The ggplot2 R package achieved the data visualization for positive TF regulators, and the ggvenn R package generated the Venn diagram.

### Pseudotime trajectory analysis

To understand the dynamic changes of brain myeloid cells’ differentiation in the uninfected and infected brains, we performed pseudo-time analyses using the Monocle^74^ implementation of ArchR. The harmony-corrected ArchR object of the core analysis was subjected to the getMonocleTrajectories and addMonocleTrajectory functions for the unsupervised trajectory analysis. In this analysis, all the myeloid cell clusters identified were used, and the root node was set as the Micro_1 cluster or CAM_1 cluster for prediction. Then, we performed the analyses for the four clusters mainly found in infected brains using the addTrajectory function, which followed a user-defined trajectory to guide the supervised trajectory analysis from the “Micro_like” to the “CAM_like_2” cluster. To visualize the changes in RNA expression, predicted gene activity, peak accessibility, and ChromVAR deviation scores across pseudo-time, we used the getTrajectory to retrieve the corresponding matrix and used the plotTrajectoryHeatmap functions to plot. Then, we performed integrative pseudo-time analyses to identify positive TF regulators driving the differentiation. This was achieved by integrating predicted gene activity or gene expression with motif accessibility across pseudo-time using the correlateTrajectories function. The TF motif database used for those correlations was JASPAR.

### Analyses for SIV infected cells

To better understand the infected myeloid cell population, we subsetted the SIV+ and SIV- cells. The SIV+ cells were defined as the cells with at least one SIV RNA transcript and one DNA fragment found, and the SIV− cells were defined as the cells with neither SIV RNA transcript nor DNA fragment found from the two SIVE animals, this resulted in 1113 SIV+ cells and 3920 SIV− cells. Then, we redid the dimensionality reduction without the SIV genome (set the excludeChr in the addIterativeLSI function to the SIV genome) for RNA expression and DNA fragments separately. We also redid the batch effect correction, UMAP, and clustering with the combined dimensionalities for the subset dataset. After getting the new cluster (6 clusters) information for each cell, we compared it with the previous annotations using the pheatmap R package. To find the upregulated and downregulated genes in more susceptible clusters, we set the useGroups in the getMarkerFeatures function as C1 and C6 clusters and set the bgdGroups as C2, C3, C4, and C5 clusters. On the other hand, we set the useGroups as C3, C4, and C5 clusters and bgdGroups as C1, C2, and C6 clusters to find the differentially expressed genes in less susceptible clusters. The statistical test method and cut-off were the same as mentioned. The detected upregulated and downregulated genes for more susceptible and less susceptible clusters were further used as input for IPA.
